# Sustained dasatinib treatment prevents early fibrotic changes following ocular trauma

**DOI:** 10.1007/s00417-020-05037-4

**Published:** 2021-01-08

**Authors:** Shunichiro Ueda, Betty M. Nunn, Rajat Chauhan, Kevin McDonald, Henry J. Kaplan, Martin G. O’Toole, Shigeo Tamiya

**Affiliations:** 1 Department of Ophthalmology and Visual Sciences, University of Louisville, 301 E Muhammad Ali Blvd, Louisville, KY 40202 USA; 2 Department of Ophthalmology, Tokyo Medical University, Tokyo, Japan; 3 Department of Bioengineering, University of Louisville, 2301 S. Third St, Louisville, KY 40292 USA; 4 Department of Ophthalmology, Saint Louis University, St. Louis, USA

**Keywords:** Ocular trauma, Fibrosis, Tyrosine kinase inhibitor, Sustained release system

## Abstract

**Purpose:**

Posterior ocular trauma and the subsequent fibrotic retinal complication termed proliferative vitreoretinopathy (PVR) are leading causes of blindness in children and young adults. A previous study suggested that changes occurring within the first month post-trauma can lead to development of PVR later. The aim of this study was to examine the effect of dasatinib, a tyrosine kinase inhibitor clinically used to treat chronic myeloid leukemia, on fibrotic changes occurring within the first month following ocular trauma.

**Methods:**

A previously established swine ocular trauma model that mimics both contusion and penetrating injuries was used. Dasatinib was administered on days 4 and 18 post-trauma via intravitreal injection of either bolus solution or suspension of a sustained release system incorporated in biodegradable poly (lactic-co-glycolic acid) (PLGA) nanoparticles. Animals were followed up to day 32, and the development of traction full-thickness fold in the posterior retina was assessed.

**Results:**

A full-thickness retinal fold extending from the wound site developed in 3 out of 4 control eyes injected with PLGA nanoparticles alone at 1 month. Administration of dasatinib solution had little preventative effect with 6 out of 7 eyes developing a fold. In contrast, dasatinib-incorporated PLGA nanoparticle injection significantly reduced the incidence of fold to 1 out of 10 eyes.

**Conclusions:**

Injection of dasatinib-incorporated PLGA significantly reduced early fibrotic retinal changes which eventually lead to PVR following posterior ocular trauma. Thus, our sustained dasatinib release system can potentially be used to both prevent and/or broaden the surgical treatment window for PVR.

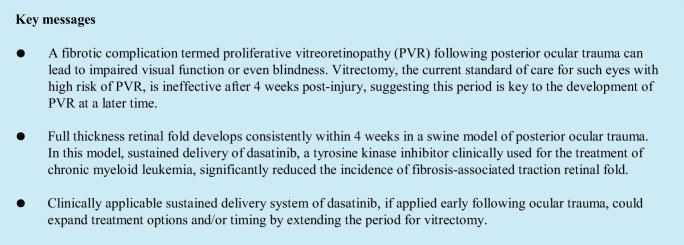



## Introduction

Ocular trauma is a leading cause of visual impairment. In particular, penetrating and perforating trauma involving the posterior segment has a high incidence of blindness [1]. Proliferative vitreoretinopathy (PVR) is the main complication leading to loss of visual acuity with incidence ranging from 10 to 50% in penetrating/perforating injuries [2, 3]. Early studies using animals, as well as clinical observation of patients, demonstrated that the vitreous plays a key role in the development of post-traumatic PVR [4, 5]; thus, vitrectomy is the current standard of care following posterior ocular trauma and associated retinal detachment as well as non-clearing vitreous hemorrhage. However, there is still a debate on the suitable timing of vitrectomy and some patients may not have access to operating facilities immediately. In a rabbit penetrating trauma model, delaying vitrectomy for 4–6 weeks had a higher risk of developing PVR when compared to vitrectomy performed within a week following injury [6]. Furthermore, vitrectomy conducted after 28 days post-injury was identified as a high risk factor for developing advanced PVR (Grade C) in patients [2], and many retinal surgeons recommend surgical intervention within 2 weeks of injury. This suggests that changes occurring within this 4-week period can lead to development of PVR at a later time point. Adjunctive therapy has been suggested to reduce the incidence of post-traumatic PVR but only a handful of agents have been tested [7–9], and currently no therapeutic agent is used routinely for this purpose.

In this study, we tested dasatinib, a tyrosine kinase inhibitor used for the treatment of chronic myeloid leukemia, as a potential adjunctive therapy to prevent fibrotic changes occurring within 4 weeks following posterior ocular trauma. Dasatinib prevents traction retinal detachment in a swine PVR model that mimics rhegmatogenous retinal detachment [10], and in vitro studies using cultured retinal pigment epithelial cells and Müller glia cells showed that dasatinib targets matrix contraction, a characteristic cellular function associated in PVR [10, 11]. We created a sustained release system for dasatinib by incorporation into poly (lactic-co-glycolic acid) (PLGA) nanoparticles (Das-PLGA) [12]. In this study, using a swine model of posterior ocular trauma [13], we examined the effect of Das-PLGA on post-injury fibrosis. We observed that intravitreal injection of Das-PLGA prevented fibrotic retinal changes within the initial 4-week period following posterior ocular trauma, thus providing a window for surgical correction of this retinal complication if it subsequently developed.

## Materials and methods

### Production and characterization of PLGA and dasatinib-incorporated PLGA particles

**Fig. 1:**
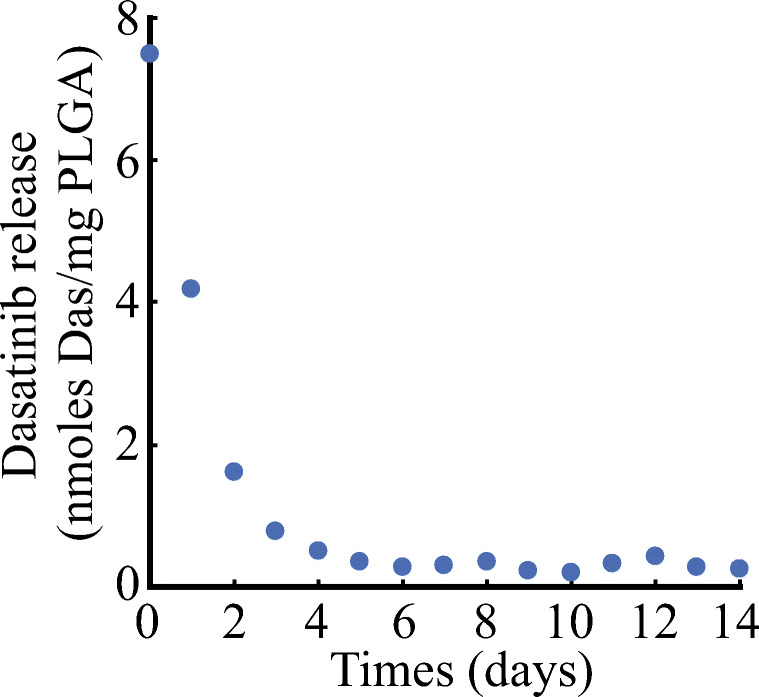
Dasatinib release profile in vitro from dasatinib-incorporated PLGA particles. Average release profile from four different batches is shown

Production of PLGA and Das-PLGA particles by spray drying method as well as characterization of particle size and release profile has been described in detail elsewhere [12]. The size of particles used for the study had an average diameter of 0.79 μm, and in vitro release profile is shown in Fig. 1.

### Full-field electroretinography measurement to determine potential adverse effects of Das-PLGA particles

PLGA is biocompatible and its intraocular use is generally well tolerated [14]. A recent study has, however, demonstrated endophthalmitis following intravitreal injection of PLGA particles [15]. Thus, full-field electroretinography (ERG) was utilized to assess potential retinal toxicity of PLGA and Das-PLGA injected intravitreally into swine eyes. The use of animals was approved by the University of Louisville Institutional Animal Care and Use Committee and adhered to the ARVO statement on the Use of Animals in Ophthalmic and Vision Research. Full-field ERG, following the ISCEV standard, was performed on swine as previously described using a UTAS ERG system with a BigShot Ganzfeld Stimulator (LKC Technologies Inc., Gaithersburg, MD) [10]. Intravitreal injection of PLGA (without dasatinib) or Das-PLGA was performed immediately after the ERG measurement (day 0, 0.5 mg particles/eye). Particle injection was repeated on day 14 but at half the amount (0.25 mg particles/eye) to reduce any possibility of adverse effects. Full-field ERG measurements were repeated on day 28 to assess the effect of particles on retinal function.

### Induction of ocular trauma in swine

Combined ocular contusion/penetrating injuries were induced based on a previously published protocol [13] with some modification. For sedation and induction of anesthesia, a combination of atropine, ketamine, butorphanol, and dexmedetomidine was administered IM, and anesthesia was maintained by isoflurane following intubation. After preparation of a conjunctival flap, contusion injury was produced nasal to the vortex veins, and 6 mm posterior to the limbus with a BB pellet. Subsequently, a fornix-based triangular scleral flap consisting of an incision 6 mm long (3 mm length on each side) penetrating sclera, choroid, and retina was created at the site of impact. Approximately 0.7 ml of prolapsed vitreous was trimmed and removed by Weck sponges, and the wound closed by suturing the apex of the triangular flap, as well as the center of each 3 mm incision. Finally, 0.7 ml of autologous blood was injected into the vitreous through the pars plana.

Bilateral surgery was approved by IACUC based on the following reasons. Published data showed, and confirmed by a preliminary study, that the outcome of retinal changes within the experimental period are limited to development of full-thickness fold in the inferior retina between the wound site and optic disc. As the visual streak of swine is superior to the optic disc, the effect of retinal fold to vision of animals was considered to be minor. Furthermore, haziness caused by blood injection was temporary, and did not cause blinding of animals that prevented basic functions such as eating or socializing. Nonetheless, animals were monitored daily, and carefully examined upon showing signs of pain or distress.

### Drug administration and evaluation of fibrotic contraction

Post-operative ocular examination involving slit lamp and indirect ophthalmoscopy, and/or B-scan ultrasound, were performed on days 4, 18, and 32. Following ocular examination on day 4, 0.1 ml of (1) PLGA particle suspension (control, without dasatinib, 0.5 mg total particles), (2) dasatinib solution (12 μM stock concentration), or (3) dasatinib-incorporated PLGA particle suspension (Das-PLGA, 0.5 mg total particles) were injected into the vitreous. Injection of 0.1 ml PLGA particle suspension (with or without dasatinib, containing 0.25 mg particles) or dasatinib solution (12 μM stock concentration) was repeated on day 18 after ocular examination. PLGA particles were halved for the second injection to avoid the possibility of vitritis previously reported by others.^14^ Animals were euthanized following the final examination (day 32), and eyecups created from unfixed enucleated eyes. Both observations from indirect ophthalmoscopy (prior to euthanasia) and examination of enucleated eyecups were used to determine development of a full-thickness fold and/or retinal detachment posterior to the wound site.

### Histological procedure

Following examination, eyecups were fixed overnight with 4% paraformaldehyde, dehydrated through a graded series of ethanol followed by xylene, and embedded in paraffin. Paraffin sections were stained for Hematoxylin and eosin (HE) (Surgipath H&E kit, Leica Biosystems, Buffalo Grove, IL), Masson’s trichrome (MT) (Artisan Masson’s trichrome staining kit, Agilent, Santa Clara, CA), and elastic van Gieson (EVG) stain using a Leica automated system (Leica Biosystems). Immunohistochemical staining for tenascin-C (TNC) and alpha-smooth muscle actin (aSMA) were performed using rabbit monoclonal antibody clone EPR4219 (Abcam, Cambridge, MA) and mouse monoclonal antibody clone ASM-1 (Leica Biosystems), respectively, and BOND polymer refine red detection system (Leica Biosystems).

### Statistical analyses

Statistical analyses were performed using Prism version 6 software. Student’s *t* test was used to compare ERG b-wave amplitude and latency time prior to and after PLGA particle injection. Fisher’s exact test was used to compare difference in number of eyes that developed full-thickness retinal folds among different treatment groups.

## Results

### ERG response in eyes injected with PLGA or Das-PLGA particles

**Fig. 2:**
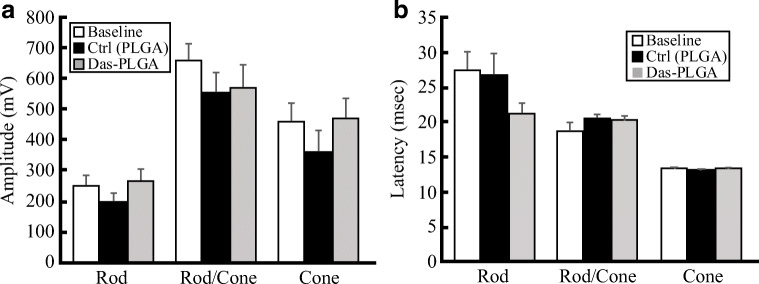
ERG response from eyes at baseline (day 0, prior to injection) and 4 weeks following injection of PLGA (control) or Das-PLGA particles (*n* = 6 each). Rod, rod/cone mix, and cone response amplitude (**a**) and latency time (**b**) (both measured from the trough of a-wave to peak of b-wave) is shown. No significant change was detected

Injection with either PLGA or Das-PLGA particles (*n* = 6 each) did not significantly affect full-field ERG responses. Average b-wave amplitudes, as well as latency, in dark-adapted 0.01 and 3 cd•s•m^−2^ (rod and rod/cone responses, respectively) and light-adapted 3 cd s m^−2^ (cone response) ERG responses measured on day 28, after injections on day 0 and 14, were not significantly different than baseline (on day 0, Fig. 2). Since there was no evidence of an adverse effect, PLGA and Das-PLGA particles were tested in our ocular trauma model.

### Early fibrotic changes following posterior ocular trauma, and effect of treatment

**Fig. 3:**
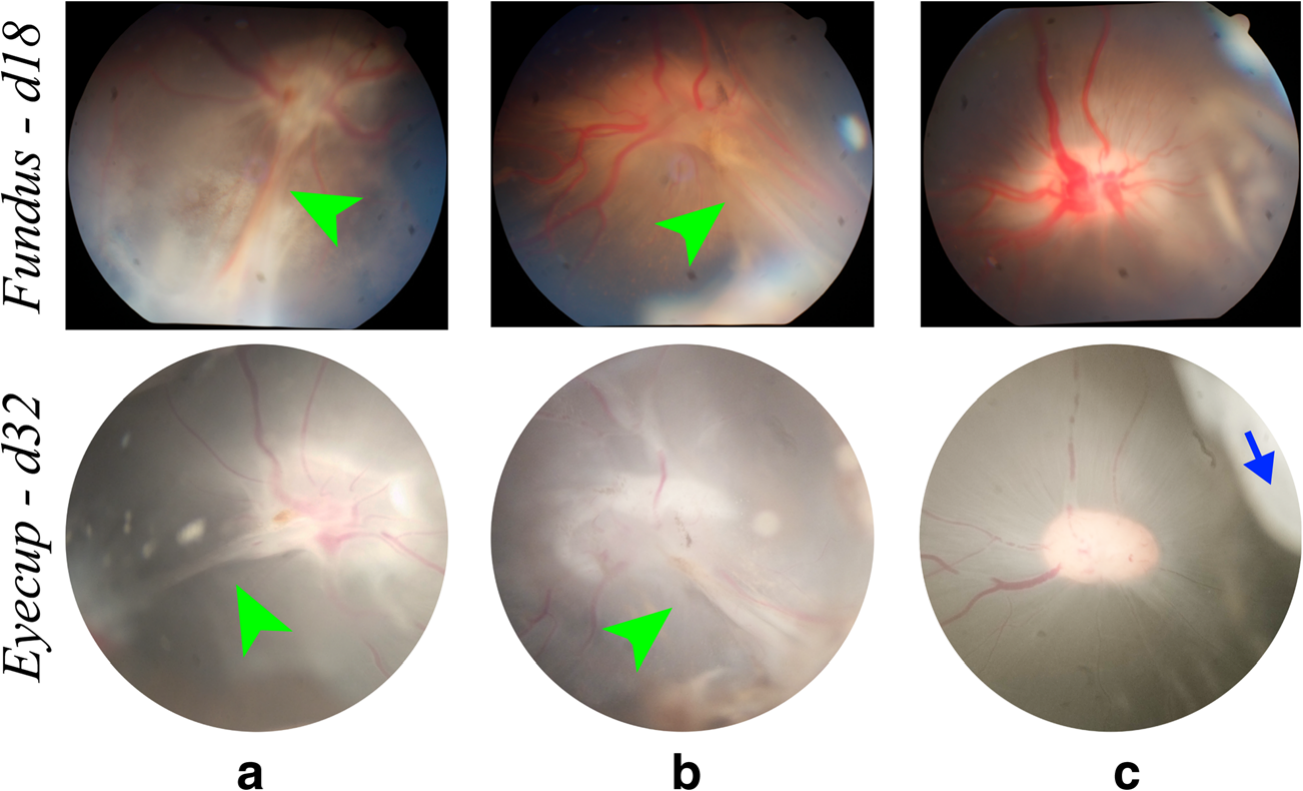
Representative fundus photographs (day 18) and eyecup images (day 32) in our swine ocular trauma model. Full-thickness posterior retinal folds (indicated by green arrowheads) develop in control eyes (injected with PLGA) (**a**) and eyes injected with dasatinib solution (Das-solution) (**b**). In contrast, full-thickness retinal fold does not develop in eyes injected with Das-PLGA (**c**). The blue arrow indicates a clump of injected Das-PLGA particles that are responsible for the artifact in the color photo

A full-thickness posterior retinal fold (Fig. 3a) developed in 3 of 4 control eyes injected with PLGA particles (Table 1). Retinal folds were observed at day 18 and remained until final examination on day 32 (Fig. 3a).

**Table 1:** Effect of treatment on development of full-thickness fold

	*n* number	Ctrl (PLGA)	Das-solution	Das-PLGA
		4	7	10
Full-thickness fold	Yes	3 (75%)	6 (86%)	1 (10%)*
	No	1 (25%)	1 (14%)	9 (90%)

*Significantly different (*p* < 0.05) from control (PLGA)

Injection of dasatinib solution (Das-solution) every other week did not prevent retinal fibrotic changes with 6 of 7 eyes developing a full-thickness posterior retinal fold (Fig. 3b, Table 1). In contrast, intravitreal Das-PLGA significantly reduced the incidence of retinal fibrosis with only 1 of 10 eyes developing a full-thickness retinal fold (Table 1). Most eyes injected with Das-PLGA had no detectable change in the posterior retina (Fig. 3c).

One eye developed signs of sterile endophthalmitis following injection of Das-PLGA. This eye was treated with intravitreal dexamethasone, and removed from the study before its completion.

### Histological analyses of the wound site

**Fig. 4:**
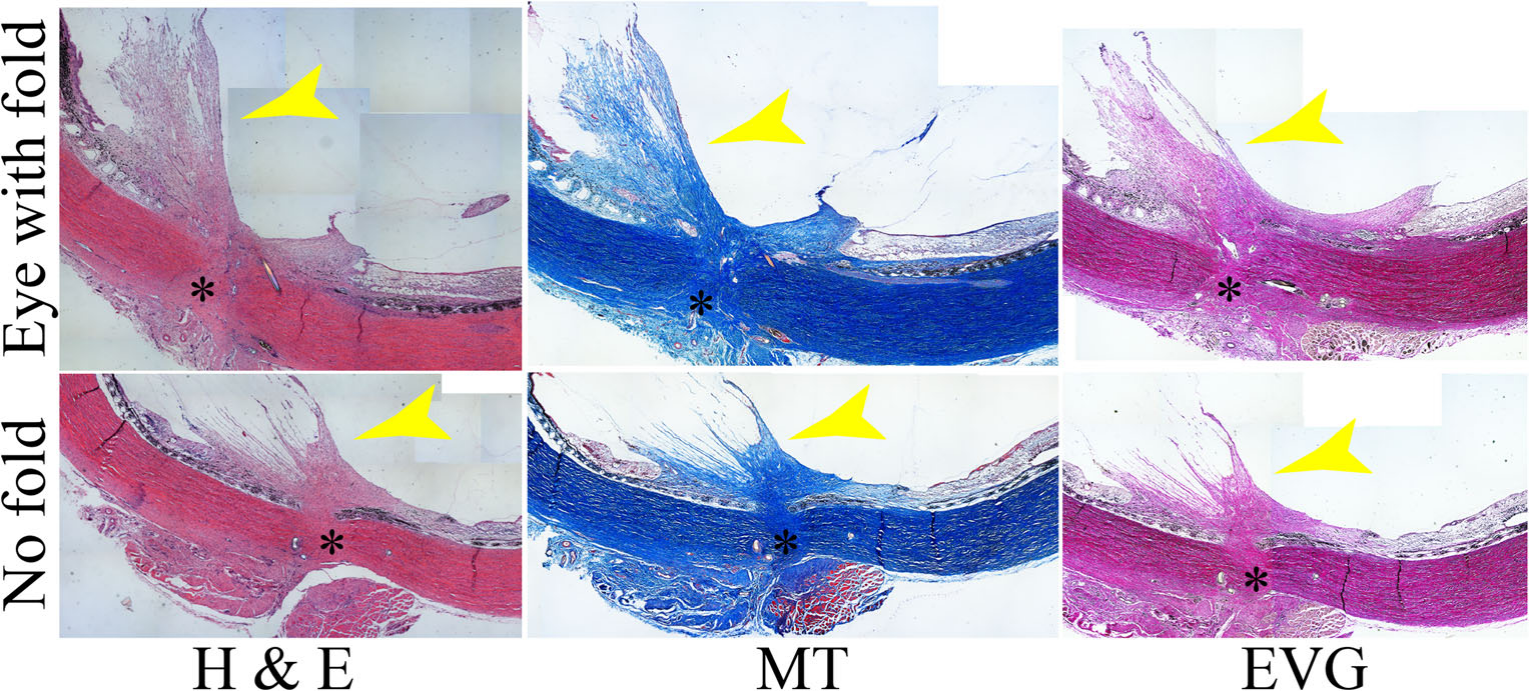
Examples of the histological sections of the wound site on day 32 . Staining with hematoxylin and eosin (H & E, left), Masson’s trichrome (MT, middle) and Elastic van Gieson (EVG, right) stain is shown. Yellow arrowheads indicate fibroproliferative scars that extend intraocularly from the wound site (*). MT and EVG stain both show strong staining for collagen (blue for MT, red for EVG) of the scar, irrespective of the development of full-thickness fold in the eye

Past studies have shown that the fibrotic proliferation at the entrance site in penetrating injuries results in vitreoretinal traction [4, 5, 16]. H&E, Masson’s trichrome (MT), and Elastic van Gieson (EVG) staining all showed inward extension of a fibroproliferative scar from the wound site (Fig. 4). MT and EVG staining confirmed strong expression of collagens (blue for MT, red for VEG) in scar tissue (Fig. 4). Incidence of scar formation on the retinal surface matched PVR grade in most eyes injected with PLGA or Das-solution (Table 2). Despite the lack of full-thickness retinal folds in 9 of 10 eyes injected with Das-PLGA, scar formation was also observed on the retinal surface in 6 out of 9 eyes (Table 2). We could not identify the wound site in one of the Das-PLGA eye due to damage to the tissue during processing, and thus, this eye was omitted from histological analyses.

**Table 2:** Summary of histological findings

	Ctrl (PLGA)	Das-solution	Das-PLGA
Retinal scar formation	3/4 (75%)	7/7 (100%)	6/9 (67%)
Tenascin-C positive scar	2/4 (50%)	3/7 (43%)	4/9 (44%)
Alpha-SMA positive scar	3/4 (75%)	6/7 (86%)	3/9 (33%)

**Fig. 5:**
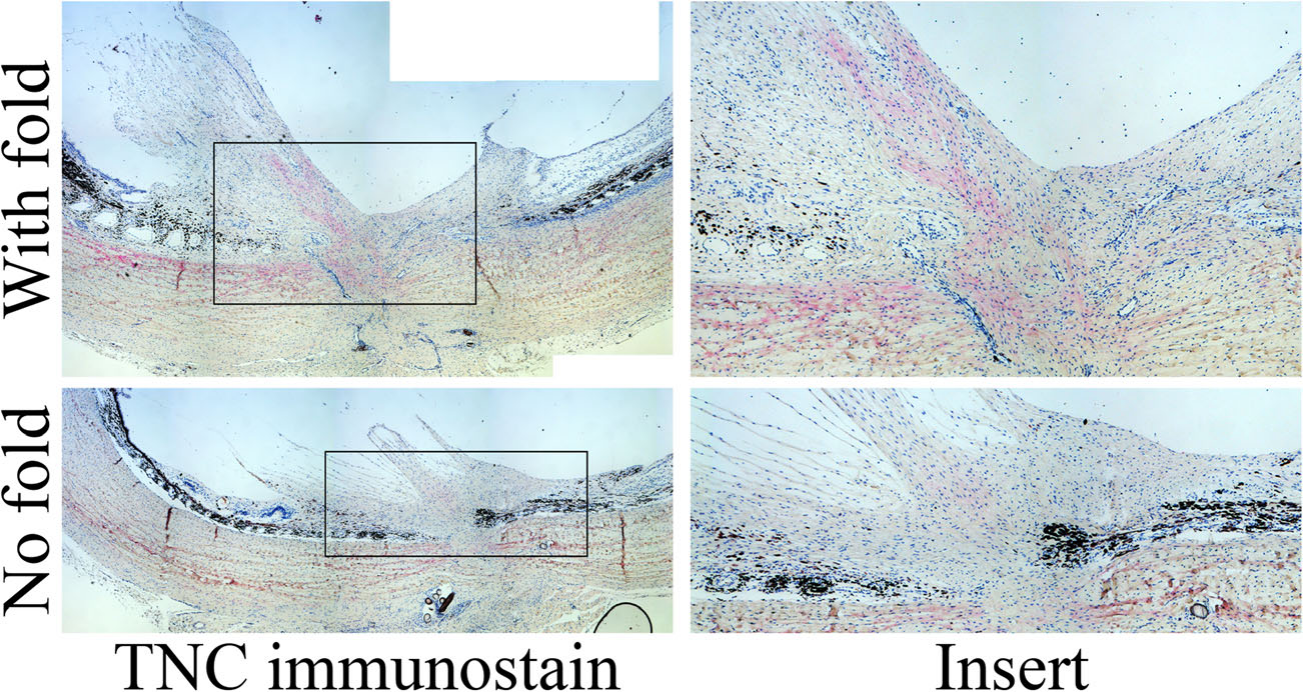
Immunohistochemical staining of tenascin-C (TNC). Top: Strong positive stain (red) is observed at the base of the scar in the eye that developed a full-thickness retinal fold. Bottom: A weaker but detectable staining for TNC is observed in the same region of the scar in the eye that did not develop a fold following Das-PLGA intravitreal injection on day 4 post-trauma

**Fig. 6:**
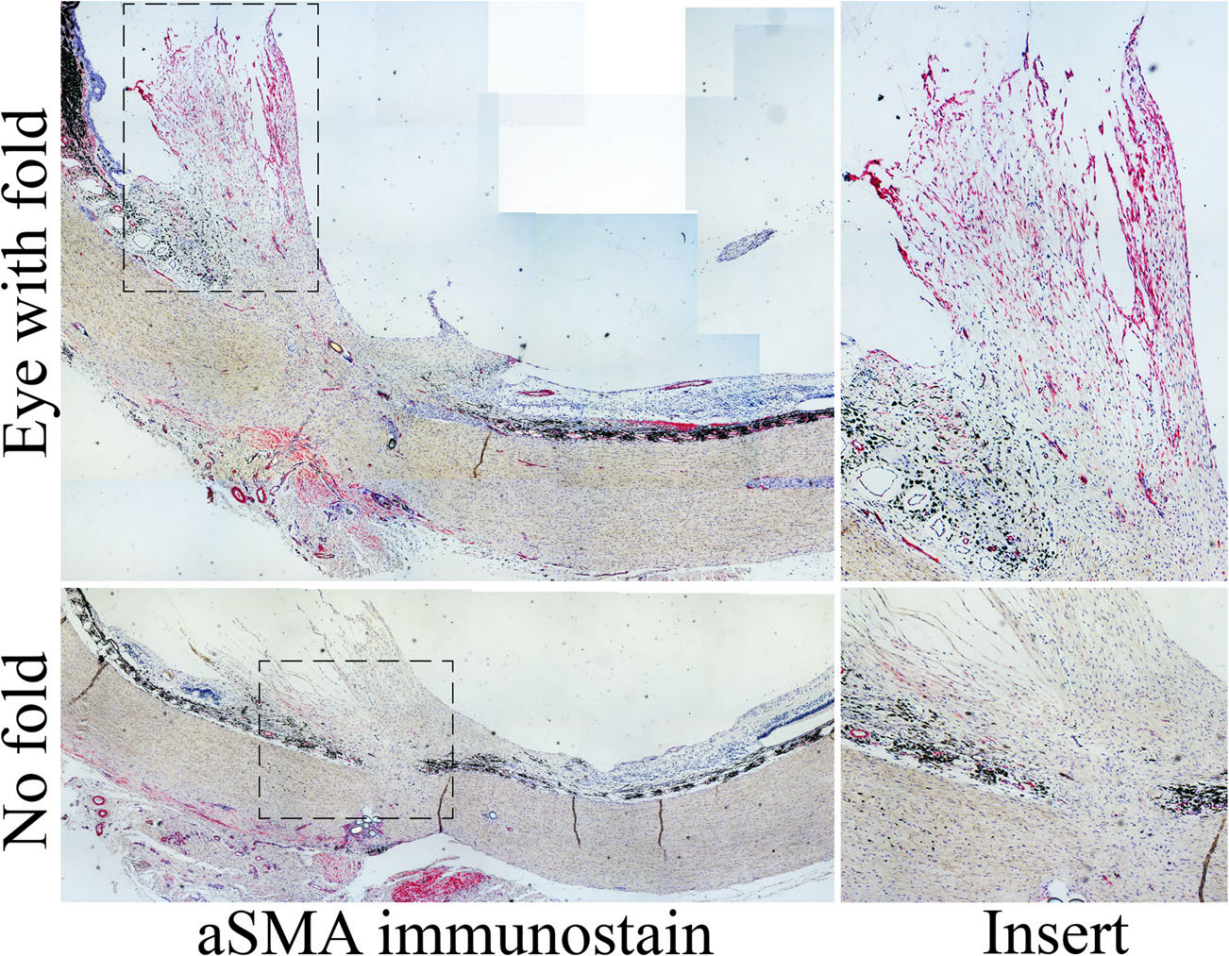
Immunohistochemical staining of alpha-smooth muscle actin (aSMA). Top: Strong signal for alpha-smooth muscle actin (red) is detected in the eye that develops a full-thickness retinal fold. Bottom: Weak/no signal for aSMA is observed in the eye that did not develop a fold following Das-PLGA intravitreal injection on day 4 post-trauma

Expression of tenascin-C (TNC) (Fig. 5), an extracellular matrix protein upregulated in various fibrotic diseases/complications, was detected by immunohistochemistry in 45% of all eyes used in the study (Table 2). Control showed noticeable expression in 2/4 eyes, and treatment with Das-solution (3/7 eyes) or Das-PLGA (4/9 eyes) had no noticeable effect on TNC expression (Table 2). Expression of aSMA (Fig. 6), a marker for myofibroblasts, matched the PVR grade in most eyes injected with PLGA (control) or Das-solution (Table 2). Eyes injected with Das-PLGA showed a trend for reduced expression of aSMA but the difference did not reach statistical significance (Table 2).

## Discussion

PVR is the major complication following posterior ocular trauma that leads to visual disability and frequently blindness. The incidence of post-traumatic PVR depends on a variety of factors such as type of injury (i.e. blunt, penetrating, perforating or rupture), presence of intraocular hemorrhage, retinal tear or retinal detachment [2, 3]. Ryan and colleagues established several animal models to study penetrating trauma [13, 17, 18], and together with observations from clinical specimens [5], concluded that fibroproliferative changes following penetrating eye injuries cause vitreous traction resulting in retinal folds and/or detachment. In this study, we adopted the swine model established by Gregory and Ryan that involves both contusion and penetrating injury, and displays fibrotic changes within 4 weeks post-injury [13]. While we shifted the injury site posteriorly from the pars plana, as in the original study, to include damage to the retina, this difference in the position of the entrance wound did not alter the outcome. Both the current study, as well as the original study [13], resulted in comparable localized, full-thickness posterior retinal folds. Furthermore, the reported time course of retinal fold development (> 8 days) [13] and the incidence of retinal folds at 2 weeks (7 out of 9 eyes) [19] of the original studies were similar to the current study. Despite penetrating injury to the retina/RPE, extensive retinal detachment did not develop in the current study. This may be due to efficient pumping function of swine RPE cells, as evidenced by reattachment of the retina within 3 days of an artificially created total retinal detachment [20], and a viscous vitreous in young swine compared to man.

Injection of Das-PLGA prevented post-traumatic fibrotic changes in the current study. Dasatinib was originally developed as a dual inhibitor of Src family kinases (SFKs) and Abl kinase [21]. However, it has subsequently been shown to inhibit a host of tyrosine kinases including PDGF receptor kinase and c-kit [22]. Dasatinib has been shown to suppress fibrotic changes in various animal models of tissue fibrosis [23–27]. Within the eye, we have previously demonstrated that dasatinib inhibits traction retinal detachment in a swine PVR model involving RPE cell injection [10]. Prevention of RPE cell epithelial-mesenchymal transition, proliferation, and migration by dasatinib was due to inhibition of SFKs [10]. Furthermore, dasatinib significantly reduced matrix contraction by cultured Müller glia cells via targeting SFKs and PYK2, both of which play a key role in phosphorylation of focal adhesion proteins [28]. While similar molecular mechanisms may be playing key roles in the prevention of fibrotic changes observed in the current study, further investigation is required for confirmation.

In the current study, bolus injection of dasatinib solution failed to inhibit the development of a retinal traction fold. This is in contrast to our previous study in which traction retinal detachment was prevented in a model that mimics PVR following rhegmatogenous retinal detachment by bolus injection of dasatinib solution. However, we injected dasatinib solution twice-a-week in that study [10], whereas in the current study it was injected every other week. Retention of small molecules such as dasatinib in the vitreous cavity is known to be relatively short, and it is likely that injection performed every other week failed to maintain an effective concentration of the drug. The fact that Das-PLGA, which releases dasatinib up to 14 days [12], was effective in preventing development of traction retinal folds in our current model strongly implies that sustained presence of the drug is required. Maintaining effective concentration is an important factor for prevention of PVR with other drugs as well. Recent studies using methotrexate for the prevention of recurrent PVR demonstrate that, while a single injection at the time of corrective surgery failed to show statistically significant difference in retinal re-detachment rate compared to control [29], no re-detachment was observed in two separate pilot studies when drug presence was sustained in eyes by repeated intraocular injection [30, 31].

PLGA is well established as a biocompatible and biodegradable polymer [14]. Multiple studies have tested PLGA for intravitreal drug delivery, and most studies have demonstrated it to be well tolerated. Our data agrees with such studies with full-field ERG not detecting any significant difference in baseline retinal function after PLGA or Das-PLGA injection. In the current study, however, we did lose one eye to endophthalmitis following Das-PLGA injection. A recent study demonstrated the effect of shape of PLGA particles on the development of intraocular uveitis, with rod-shaped PLGA implants being innocuous compared to PLGA microparticles that induced ophthalmitis [15]. Furthermore, PLGA nanoparticles that were about half the diameter of particles used in this study were superior to PLGA microparticles in intraocular delivery of EGF receptor tyrosine kinase inhibitor AG1478 [32]. Therefore, further testing and optimization of the shape and size of PLGA for intravitreal drug delivery may be warranted.

In summary, intravitreal injection of Das-PLGA significantly inhibited development of traction retinal folds, which could eventually lead to severe PVR, in our pig model of posterior intraocular traumatic injury. Vitrectomy is used to repair posterior ocular trauma but its optimal timing remains a source of debate [33, 34]. Prevention or even significant delay of early fibrotic changes will allow more treatment options, as well as broadening the timing of surgical intervention. Our data suggests that adjunctive therapy with Das-PLGA shortly after posterior segment traumatic wounds will minimize early fibrotic changes and that sustained dasatinib release systems merit further investigation to prevent the loss of vision associated with PVR.
